# Supplementary material for: Principles of operation of a cerebellar learning circuit
Source: eLife. 2020 Apr 30;9:e55217. doi: 10.7554/eLife.55217 (PMC7255800; doi:10.7554/eLife.55217)
Supplement: Transparent reporting form [file elife-55217-transrepform.pdf]

eLIFE
Westbrook Centre, Milton Road
Cambridge CB4 1YG
UK
P 01223 855340
W elifesciences.org
T @elife

eLife's transparent reporting form

We encourage authors to provide detailed information *within their submission* to facilitate the interpretation and replication of experiments. Authors can upload supporting documentation to indicate the use of appropriate reporting guidelines for health-related research (see EQUATOR Network), life science research (see the BioSharing Information Resource), or the ARRIVE guidelines for reporting work involving animal research. Where applicable, authors should refer to any relevant reporting standards documents in this form.

If you have any questions, please consult our Journal Policies and/or contact us: editorial@elifesciences.org.

Sample-size estimation

- You should state whether an appropriate sample size was computed when the study was being designed
- You should state the statistical method of sample size computation and any required assumptions
- If no explicit power analysis was used, you should describe how you decided what sample (replicate) size (number) to use

Please outline where this information can be found within the submission (e.g., sections or figure legends), or explain why this information doesn't apply to your submission:

Replicates

All data shown in the manuscript were collected from two male rhesus monkeys (first sentence of Materials and Methods). Each experiment was performed across multiple days/sessions. Data using non-human primates are typically performed on n=2 subjects. No explicit power analysis was performed to determine the number of primates necessary for each experiment.

Each experiment was repeated on each animal multiple times (behavioral session/days). Information regarding the number of session is found in the replicates section, below, as well as in the main text associated with each experiment. All statistical analyses are performed separately for each monkey where statistics were computed across individual sessions/learning blocks. When at all possible, we show individual data for each monkey separately.

- You should report how often each experiment was performed
- You should include a definition of biological versus technical replication
- The data obtained should be provided and sufficient information should be provided to indicate the number of independent biological and/or technical replicates
- If you encountered any outliers, you should describe how these were handled
- Criteria for exclusion/inclusion of data should be clearly stated
- High-throughput sequence data should be uploaded before submission, with a private link for reviewers provided (these are available from both GEO and ArrayExpress)

eLife Sciences Publications, Ltd is a limited liability non-profit non-stock corporation incorporated in the State of Delaware, USA, with company number 5030732, and is registered in the UK with company number FC030576 and branch number BR015634 at the address Westbrook Centre, Milton Road Cambridge CB4 1YG, UK | March 2019

Please outline where this information can be found within the submission (e.g., sections or figure legends), or explain why this information doesn't apply to your submission:

No data/outliers were removed from the results. All data were measured from both monkeys were included in the results/analysis provided the animal completed the trial (see Materials and Methods, subsection: Behavioral procedures, for information regarding conditions in which the trial was aborted).

Each experimental condition was performed multiple times for each monkey. Statistical analysis and summary figures are provided for each animal separately (where possible). The number of replicates for each animal for experimental condition (typically greater than 5) is provided in the text as the degrees of freedom in the statistical calculations.

Statistical reporting

- Statistical analysis methods should be described and justified
- Raw data should be presented in figures whenever informative to do so (typically when N per group is less than 10)
- For each experiment, you should identify the statistical tests used, exact values of N, definitions of center, methods of multiple test correction, and dispersion and precision measures (e.g., mean, median, SD, SEM, confidence intervals; and, for the major substantive results, a measure of effect size (e.g., Pearson's r, Cohen's d)
- Report exact p-values wherever possible alongside the summary statistics and 95% confidence intervals. These should be reported for all key questions and not only when the p-value is less than 0.05.

Please outline where this information can be found within the submission (e.g., sections or figure legends), or explain why this information doesn't apply to your submission:

(For large datasets, or papers with a very large number of statistical tests, you may upload a single table file with tests, Ns, etc., with reference to sections in the manuscript.)

We outline our statistical tests/statistical approach in the Materials and Methods section (subsection: Statistical analysis). As noted above, statistical tests were performed separately for each monkey where possible. We report not only the statistical test performed and the resulting exact p-value, but also the complete degrees of freedom for each test as well as the test statistic. These statistical descriptions provide the exact number (N) of replicates performed for each monkey and each experimental condition.

All individual tests were first confirmed by an omnibus ANOVA, eliminating the need to performed post-hoc corrections to the statistical analysis.

The errorbars and errorbands for all figures are defined in their respective figure legends (typically mean +/- SEM across experimental sessions). Reports of individual values fitted to the data are reported as mean +/- 95% CI, as noted in the text.

Group allocation

- Indicate how samples were allocated into experimental groups (in the case of clinical studies, please specify allocation to treatment method); if randomization was used, please also state if restricted randomization was applied
- Indicate if masking was used during group allocation, data collection and/or data analysis

Please outline where this information can be found within the submission (e.g., sections or figure legends), or explain why this information doesn't apply to your submission:

Additional data files ("source data")

No randomization was used to assign monkeys to individual groups. Both monkeys completed all experimental conditions.

Randomization was only performed within experimental conditions (see Materials and Methods).

- We encourage you to upload relevant additional data files, such as numerical data that are represented as a graph in a figure, or as a summary table
- Where provided, these should be in the most useful format, and they can be uploaded as "Source data" files linked to a main figure or table
- Include model definition files including the full list of parameters used
- Include code used for data analysis (e.g., R, MatLab)
- Avoid stating that data files are "available upon request"

Please indicate the figures or tables for which source data files have been provided:

Please see section titled "Data and code availability."

Briefly, the data for each figure is included with the submission as a Figure Composer FYP file. Figure composer is a freely available open-source tool for the generation of figures. The figure composer file format is open and machine readable, allowing other users to implement their own tools to do additional data processing. The GUI associated with Figure Composer can also read, modify, and export data associated with each Figure Composer file to standard formats.

Information about Figure Composer can be found at its website:
https://sites.google.com/a/srscicomp.com/figure-composer

Source code for the simulations are attached to the submission as a Jupyter notebook. All parameters associated with the model are included in the text of the manuscript in Table 1 as well as in the uploaded source code.
